# Chemoattractant levels in nasal secretions as indicators of clinical severity in chronic polypous rhinosinusitis

**DOI:** 10.1186/2045-7022-3-S2-O19

**Published:** 2013-07-16

**Authors:** Aleksandar Peric, Danilo Vojvodic, Nenad Baletic, Vesna Radulovic

**Affiliations:** 1 Faculty of Medicine, Military Medical Academy, Department of Otorhinolaryngology, Belgrade, Serbia; 2 Faculty of Medicine, Military Medical Academy, Department of Clinical and Experimental Immunology, Belgrade, Serbia; 3 Municipal Institute for Lung Diseases, Department of Pneumology, Belgrade, Serbia

## Background

The local accumulation of activated eosinophils in the nasal mucosa is a feature of chronic polypous rhinosinusitis (CPRS). However, the pathogenesis of chronic hypereosinophilia in nasal polyps is still unknown. An increased production of several chemoattractants, responsible for guiding the inflammatory process, has been reported in this disease. The aim of this study was to evaluate nasal secretion levels of several chemokines and to correlate those levels with clinical characteristics and degree of eosinophilia in asthmatic and non-asthmatic patients with polypous sinusitis.

## Method

Fourteen non-atopic non-asthmatic patients with CPRS and 14 CPRS patients with co-morbid atopic asthma were recruited for this cross-sectional study. Fourteen healthy subjects were included as controls. The concentrations of GM-CSF, MCP-1, MCP-3, MIP-1alpha, MIP-1beta, and RANTES in nasal secretions were measured using flow-cytometric method. Eosinophil counts were performed by percentage of differential granulocyte counts during cytological examination of scraped nasal mucosa obtained from the inferior turbinate by rhinoprobe. Therefore, we scored clinically each of the 28 nasal polyp patients according to the nasal symptom score, endoscopic score, and Lund-Mackay CT score.

## Results

We found significantly higher concentrations of MCP-1 (p<0.0001), MCP-3 (p=0.018) and RANTES (p<0.0001) in nasal fluid of asthmatic CPRS patients compared to non-asthmatic. In non-asthmatic patients, we found positive correlation between MIP-1alpha levels and nasal symptom score, at the 0.01 level (p=0.01). Therefore, the concentrations of RANTES were positive correlated with nasal symptom score, nasal endoscopic score, CT score, and eosinophil counts (p=0.01). On the other hand, in asthmatic patients, the concentrations of MCP-1 were associated with nasal symptom score, nasal endoscopic score, Lund-Mackay score, and percentage of eosinophils (p=0.01). The concentration of RANTES in asthmatic CPRS patients also correlate with all clinical parameters, and with degree of eosinophilia (p=0.01).

## Conclusion

CPRS in asthmatics is characterized by higher degree of eosinophilic inflammation than in non-asthmatics. RANTES and MCP-1 levels correlate well with clinical severity of CPRS. The measurment of chemokines in nasal secretions could be useful in evaluating the degree of sinonasal inflammation in nasal polyp patients.

